# Nitrogen transformation processes catalyzed by manure microbiomes in earthen pit and concrete storages on commercial dairy farms

**DOI:** 10.1186/s40793-023-00483-z

**Published:** 2023-04-11

**Authors:** Bela Haifa Khairunisa, Usha Loganathan, Jactone A. Ogejo, Biswarup Mukhopadhyay

**Affiliations:** 1 Genetics, Bioinformatics, and Computational Biology Ph.D. Program, Virginia Tech, Blacksburg, VA 24061 USA; 2 Department of Biochemistry, Virginia Tech, Blacksburg, VA 24061 USA; 3 Department of Biological System Engineering, Blacksburg, VA 24061 USA

**Keywords:** Dairy manure, Storage, Nitrogen fertilizer, Nitrogen loss, Greenhouse gas emission, Microbiome, Metabolism, Methanogen, Methane

## Abstract

Storing manure is an essential aspect of nutrient management on dairy farms. It presents the opportunity to use manure efficiently as a fertilizer in crop and pasture production. Typically, the manure storages are constructed as earthen, concrete, or steel-based structures. However, storing manure can potentially emit aerial pollutants to the atmosphere, including nitrogen and greenhouse gases, through microbial and physicochemical processes. We have characterized the composition of the microbiome in two manure storage structures, a clay-lined earthen pit and an aboveground concrete storage tank, on commercial dairy farms, to discern the nitrogen transformation processes, and thereby, inform the development of mitigation practices to preserve the value of manure. First, we analyzed the 16S rRNA-V4 amplicons generated from manure samples collected from several locations and depths (0.3, 1.2, and 2.1–2.75 m below the surface) of the storages, identifying a set of Amplicon Sequence Variant (ASVs) and quantifying their abundances. Then, we inferred the respective metabolic capabilities. These results showed that the manure microbiome composition was more complex and exhibited more location-to-location variation in the earthen pit than in the concrete tank. Further, the inlet and a location with hard surface crust in the earthen pit had unique consortia. The microbiomes in both storages had the potential to generate ammonia but lacked the organisms for oxidizing it to gaseous compounds. However, the microbial conversion of nitrate to gaseous N_2_, NO, and N_2_O via denitrification and to stable ammonia via dissimilatory nitrite reduction seemed possible; minor quantities of nitrate was present in manure, potentially originating from oxidative processes occurring on the barn floor. The nitrate-transformation linked ASVs were more prevalent at the near-surface locations and all depths of the inlet. Anammox bacteria and archaeal or bacterial autotrophic nitrifiers were not detected in either storage. Hydrogenotrophic *Methanocorpusculum* species were the primary methanogens or methane producers, exhibiting higher abundance in the earthen pit. These findings suggested that microbial activities were not the main drivers for nitrogen loss from manure storage, and commonly reported losses are associated with the physicochemical processes. Finally, the microbiomes of stored manure had the potential to emit greenhouse gases such as NO, N_2_O, and methane.

**Supplementary information:**

The online version contains supplementary material available at 10.1186/s40793-023-00483-z.

## Introduction

The shift from family owned small dairy farms to large dairy operations in the US over the past decades has been accompanied by the generation of high volumes of manure [1, 2], and the associated accumulation and concentration of nitrogen, phosphorus, potassium, salts, and minerals in specific geographical zones [3]. The high nutrient content of manure makes it a valuable source of organic fertilizer for crops and pasture production. Thus, an effective manure management involving storage prior to application on land is an important factor driving the sustainability of dairy operations. Storing manure allows the (i) use of manure at the right time, (ii) decrease manure handling costs, and (iii) minimize the potential to pollute the environment. During storage, the organic nitrogen of manure is converted via physicochemical and microbial processes into plant available inorganic species, such as ammonia (NH_3_), nitrite (NO_2_^−^), and nitrate (NO_3_^−^) [4–7]. However, these transformations also cause the production of gaseous forms of nitrogen such as dinitrogen (N_2_), nitric oxide (NO), and nitrous oxide (N_2_O), which along with ammonia, are amenable to loss to the atmosphere unless they are rapidly converted into soluble compounds [8, 9]. Nitrogen loss from manure storage could amount to 30 percent of the total nitrogen contents depending on the storage condition [10], substantially reducing the fertilizer value of the material. Additionally, the anaerobic microbial decomposition of organic matter in manure generates methane (CH_4_), which along with NO and N_2_O are potent greenhouse gases (GHGs), making manure storage an agricultural greenhouse gas source [11]. The manure management systems contribute 9.7 percent of the methane emission in the US [12]. Thus, an understanding of the microbe-mediated nitrogen and carbon transformation in these units is necessary to develop strategies for preserving the nitrogen fertilizer value of manure and mitigating greenhouse gas emissions from these sources.

While there have been studies on these processes, the attention primarily has been on the chemical reactions mediating the losses [13, 14]. The few studies that analyzed the microbiomes of stored manure [15–21] did not focus on the role of microbes in nitrogen transformation processes but the emergence of antibiotic resistant species [16–18] and methane production [19–21]. To fill this gap, we assessed the potentials of microbial nitrogen biotransformation in a clay-lined earthen pit (EP) and an above ground concrete manure (CS) storage employing a culture-independent approach. The characteristics of the methanogens which carry out the terminal step of the biomethanation of organic materials were investigated as well. The study also tested the hypothesis that the nitrogen transformation and methanogenesis activities are influenced by the storage types.

## Materials and methods

### Storage description

**Fig. 1:**
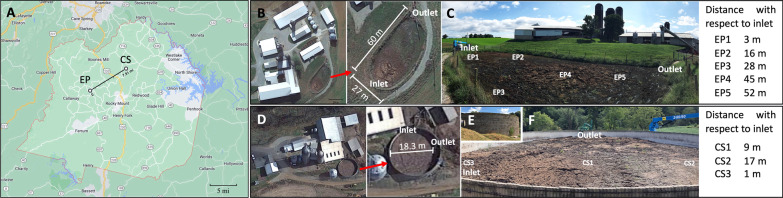
Location of manure storages studied. The locations of the earthen pit and concrete storage (A). The two storages are located 12.7 km apart. The boundary of the Franklin county is marked in red. Satellite imaging for EP (B) and CS (D). Sampling locations for EP (C): EP1, inlet with 15 cm dry crust on the surface; EP2, no crust; EP3, close to lining and no crust; EP4, with 30 cm dry crust; EP5, closest to outlet with no crust. Sampling locations for CS (E–F): CS3, inlet; CS1, middle of the storage; CS2, farthest from inlet. All sampling locations of CS had surface crust with thickness ranging from 15 to 22 cm. Map and satellite images were generated using Google Earth, https://earth.google.com/web/ on June 1, 2022

Two on-farm manure storages, a clay-lined earthen pit (EP) and a partial aboveground concrete tank (CS), were studied. The farms are located in Franklin County, VA (Fig. 1A). The EP is an earthen pit with a clay lining (Figs. 1B-C), while the CS was an aboveground tank made of concrete (Figs. 1D-E). The EP and CS received manure from 85 and 75 cows, respectively. At each farm, the cows are raised in a barn and fed a total mixed ration diet, and the manure is scraped from the barn floors to the storage twice daily.

The EP is oval, with top surface dimensions of 60 and 27 m on the long and wide sides (Fig. 1B-C). The manure inlet and outlet (pump-out) locations are on opposite sides of the longer dimension of the storage. The depth of the storage pit increases gradually from 3.66 m (near the inlet) to 3.96 m (near the outlet). The design storage capacity is enough to hold manure for about four months. For EP the manure was fed from the bottom at a location near the periphery of the pit (EP1, Fig. 1C) and for CS the addition also occurred at a periphery location but on the surface (CS3, Fig. 1F). The CS structure had a diameter of 18.3 m and was 4.6 m deep (Figs. 1D and 1E-F).

### Manure sample collection and processing

The samples were collected from the EP in August 2018 and from the CS in September 2018. At the time of the experiment, these storages were about 90% full from four months of filling, starting from an empty stage to the manure depths of 2.9 and 3.05 m for the EP and CS, respectively. In each case, the sampling locations were selected based on their distances from the inlet and outlet, and the occurrence of a typical physical structure, crust, on the surface. Samples were collected from the following five locations of EP (Fig. 1C) and three locations of CS (Fig. 1F): EP1, inlet with 15 cm dry crust; EP2, closest distance to EP1 with no crust; EP3, close to lining with no crust; EP4, 30 cm dry crust; EP5, closest to outlet with no crust; CS3, inlet; CS1, middle of the storage; CS2, farthest from inlet. The crusting profile on the surface of CS was similar at all sampling locations and ranging from 15 to 22 cm.

A self-propelled commercial telescopic boom lift with an 80 ft reach (Genie S-85, Genie United States, Redmond, WA) was used to reach a sampling location above the manure pit. Then, a custom-built sampler (Additional file 1: Fig. S1) was used to collect samples from the following three depths as measured from the surface: 0.3 m, *near-surface*; 1.2 m, *middle*; 2.1–2.7 m, *bottom*. The sampler was made of ¾ and 1 ½ inch diameter PVC pipes fitted with manually operated butterfly valve (Additional file 1: Fig. S1).

Immediately after retrieval, each sample was placed in a plastic beaker and gently mixed with a spoon; prior to their use, the beaker and spoon were washed with 2% phosphoric acid to remove contaminating nucleic acids. Then, the sample was distributed into three sterile, DNase-free 15 ml polypropylene tubes (catalog number: 62406–200, VWR International, Radnor, PA) and snap-frozen in a dry ice and ethanol bath; samples in these tubes were considered replicates. Another aliquot (~ 0.5L) of sample were placed in separate bottles to analyze for manure chemical characteristics. A total of 45 and 27 manure samples were collected from EP and CS, respectively. These were transported to the laboratory on dry ice and stored at -20 °C.

### Manure characteristics

The manure samples were analyzed for the content of total and volatile solids (TS and VS, respectively), and pH according to the standard method for wastewater analysis (APHA, 2012) as follows. The pH was measured using the IDS pH combined electrode (SenTix® 940–3, Wissenschaftlich-Technische Werkstätten GmbH, Weilheim, Germany) while the total chemical oxygen demand (COD) was analyzed using a HACH method 8000 (HACH, Colo., USA). The content of important nutrients for plant present in the manure, including total nitrogen (TN), total ammonium nitrogen (TAN), nitrate nitrogen (NO_3_^−^-N), total phosphorus, potassium, calcium, magnesium, sulfur, iron, manganese, zinc, copper, boron, molybdenum, aluminum, and sodium, were analyzed at the Agronomic Services lab, North Carolina Department of Agriculture & Consumer services (Raleigh, NC).

### DNA extraction and 16S rRNA amplicon sequencing

From a manure sample, DNA was extracted using Qiagen Fast DNA Stool Mini Kit (cat. no. 51604, Qiagen, Germantown, MD) following the manufacturer’s instructions with modification (Additional file 1: Method S1). The DNA preparations that passed quality assessment were used for paired-end sequencing targeting the 16S rRNA hypervariable region 4 (V4), using 515F and 806R primers [22, 23] at the Environmental Sample Preparation and Sequencing Facility of the Argonne National Laboratory or ANL (Lemont, IL).

### Bioinformatic analysis

The QIIME 2–2019.4 and PICRUSt2 v.2.1.4 pipelines were used on the high-performance computing cluster of the Virginia Tech Advanced Research Computing (ARC) resources. The analysis relies on sequences of a short section of the 16S rRNA gene and not whole genomes or isolate characteristics. Thus, the detected Amplicon Sequence Variants (ASVs) represent organisms that are highly similar and not identical to known archaea and bacteria that we list in the report.

#### Taxonomic and abundance analysis of the of the 16S rRNA sequences

Raw sequence data obtained from the ANL were analyzed by the QIIME 2–2019.4 package [24] for preprocessing and removal of contaminants. The ASVs were generated via DADA2 pipeline [25] and then clustered at 99% sequence similarity using vsearch [26]. A pre-trained Naïve Bayes classifier was used to annotate the sequences using the SILVA 132 database [27]. Sequences annotated as chloroplast and mitochondria were classified as contaminants [28–30] and removed from the dataset. Statistical and phylogenetic analysis were done using Bioconductor packages [31] in R [32] as follows.

Species richness index was calculated using Chao1 estimator of the microbiomeSeq package [33] with samples rarefied to 4529 sequences per sample. Significant differences between species richness of two groups were determined by pairwise ANOVA (*P*_ANOVA_ < 0.05). Microbial community comparison between samples was performed via non-metric multidimensional scaling (nMDS) ordination of the Bray–Curtis dissimilarity distances [34]. The sample parameters that contributed the most to sample clustering were identified via a non-parametric permutational analysis of variance (PERMANOVA) and analysis of similarities (ANOSIM) of adonis function in vegan [35] (permutation: 999, *P* < 0.05) [36].

The microbiome composition of stored dairy manure was assessed using phyloseq and microbiome packages [37, 38]. Prior to the analysis, the ASVs were normalized to its relative abundance. The microbial species that were more enriched in one sample group (at a location or depth) versus another were identified using differential abundance analysis on DESeq2 [39] with *P*_wald_ < 0.001, fitType = “parametric”, and sfType = “poscounts”. The significance of the difference between the abundances of Euryarchaeota members across sampling parameters, specifically *Methanocorpusculaceae*, was assessed using non-parametric Kruskal–Wallis [40] and pairwise Wilcoxon comparison test with continuity correction [41] (*P*_Kruskal-Wallis_ = 0.05 and *P*_Wilcoxon_ = 0.05).

#### Linking the ASVs to the nitrogen transformation pathways

**Fig. 2:**
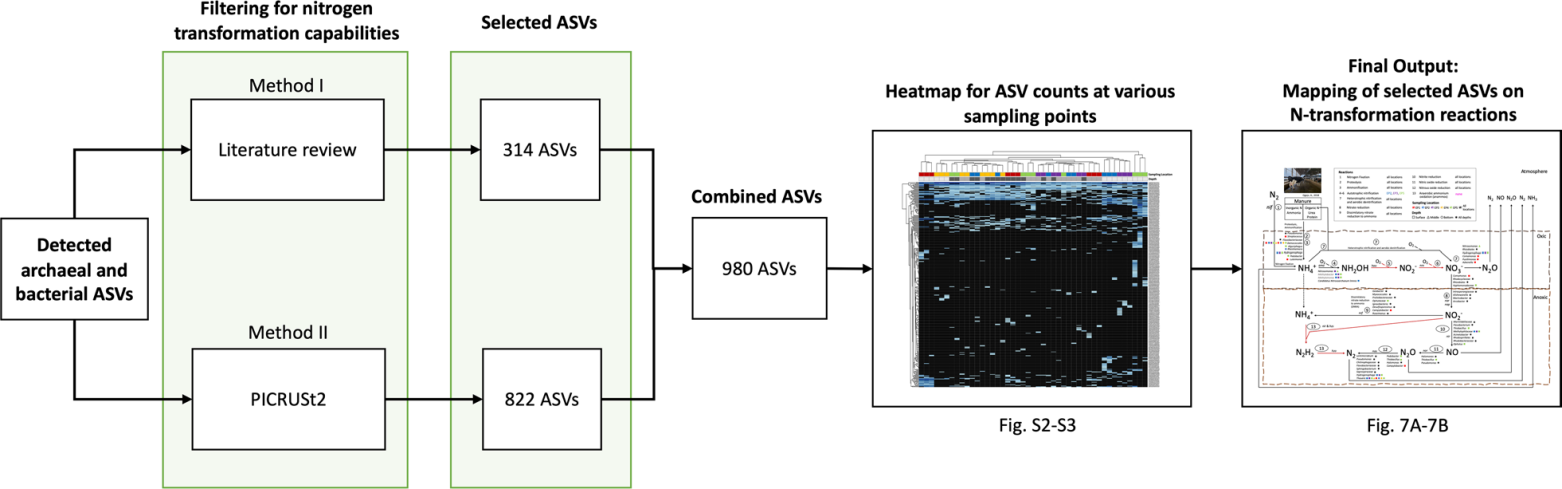
Workflow for the identification of microbial nitrogen transformation pathways operating in stored manure. A combination of initial filtering via literature review and PICRUSt2 resulted in a curated database consisting of 980 ASVs with links to nitrogen transformation processes. These annotations and the abundances of the ASVs (Additional file 1: Figs. S2 and S3) helped to build the scheme shown in Figs. 7A and B

The workflow as shown in Fig. 2 was used to assign the nitrogen (N) transformation capabilities to the detected ASVs based on their lowest valid taxonomic annotations. This analysis employed two approaches (Fig. 2), one of which was based on a literature review (Additional file 2: Table S1) and the other utilized PICRUSt2 v.2.1.4 which linked the appropriate ASVs to the nitrogen-transformation genes by leveraging available genomic libraries [42]. For PICRUSt2, the option of “metagenome_contrib in the metagenome_pipeline.py” was used to list ASVs with link to nitrogen transformation capabilities broadly (missing hydroxylamine oxidase (EC 1.7.3.6), hydrazine synthase (EC 1.7.2.7), and hydrazine oxidase (EC 1.7.2.8)), while “per_sequence_contrib option in the pathway_pipeline.py” was used to focus on denitrification [42]. The information generated using these two approaches was combined, and a heatmap for the relative abundances of ASVs linked to the N-transformation capabilities was generated using pheatmap ver 1.0.12 [43] (Additional file 1: Figs. S2 and S3). Then the predicted capabilities of the ASVs showing significant relative abundances were used to build a scheme of the potential N-biotransformation pathways by the microbiome in manure storage. This analysis revealed 13 reactions for the EP and CS manure storage structures (Figs. 7A and B).

## Results

### Manure characteristics

The analysis targeted five locations in EP and three locations in CS. The reason for this difference is that for EP the thickness of surface crust varied from location to location, whereas it was uniform for CS. The pH, COD, and the nutrient profile of the manure samples are presented in Additional file 2: Table S2. Manure sampled from 15 cm below at the location closest to the inlet (EP1-near-surface in EP, Fig. 1C; CS3-near-surface in CS, Fig. 1F) contained the highest COD, TS, VS, TN, organic nitrogen content (ORG-N), and NO_3_^−^-N levels (Additional file 2: Table S2). The same observation was also made for the TAN at EP1 but not CS3. In fact, all other samples in CS contained at least four times more TAN than the near-surface samples collected at the inlet (CS3-near-surface; Fig. 1F and Additional file 2: Table S2). Another unusual observation was that in the EP, the next highest levels of TS, VS, total Kjeldahl nitrogen (TKNTN), ORG-N, and TAN were found at EP4 which was located halfway between the inlet and the outlet (Fig. 1C); this location however did not have a high level of NO_3_^−^-N. The observed very high TS, VS, COD, TKNTN, ORG-N, NO_3_^—^N, TAN, and other nutrient values in the CS3-near-surface sample could be an artifact caused a constituent such as a lump of feces. Within EP, the lowest pH value (6.92) was found in the EP1-near-surface sample, whereas the EP3-middle and EP4-near-surface exhibited the highest values of 7.66 and 7.85 (Additional file 2: Table S2), respectively. Except for pH and NO_3_^−^-N content, the nutrient-rich features of EP1-near-surface and EP4-near-surface were also observed in EP1-bottom and EP4-bottom locations. Some of the samples taken from the middle depth, especially those from EP2, EP3, and EP5 locations, showed the lowest organic matter concentrations (Additional file 2: Table S2).

### 16S rRNA-V4 amplicon sequences of stored dairy manure samples

Sequencing of the 16S rRNA-V4 region of the DNA preparations generated 872,408 sequences with 3,719 ASVs. Clustering of the ASVs at the 99% similarity threshold produced 872,194 reads with 2,885 ASVs.

### Species richness in stored dairy manure

**Fig. 3:**
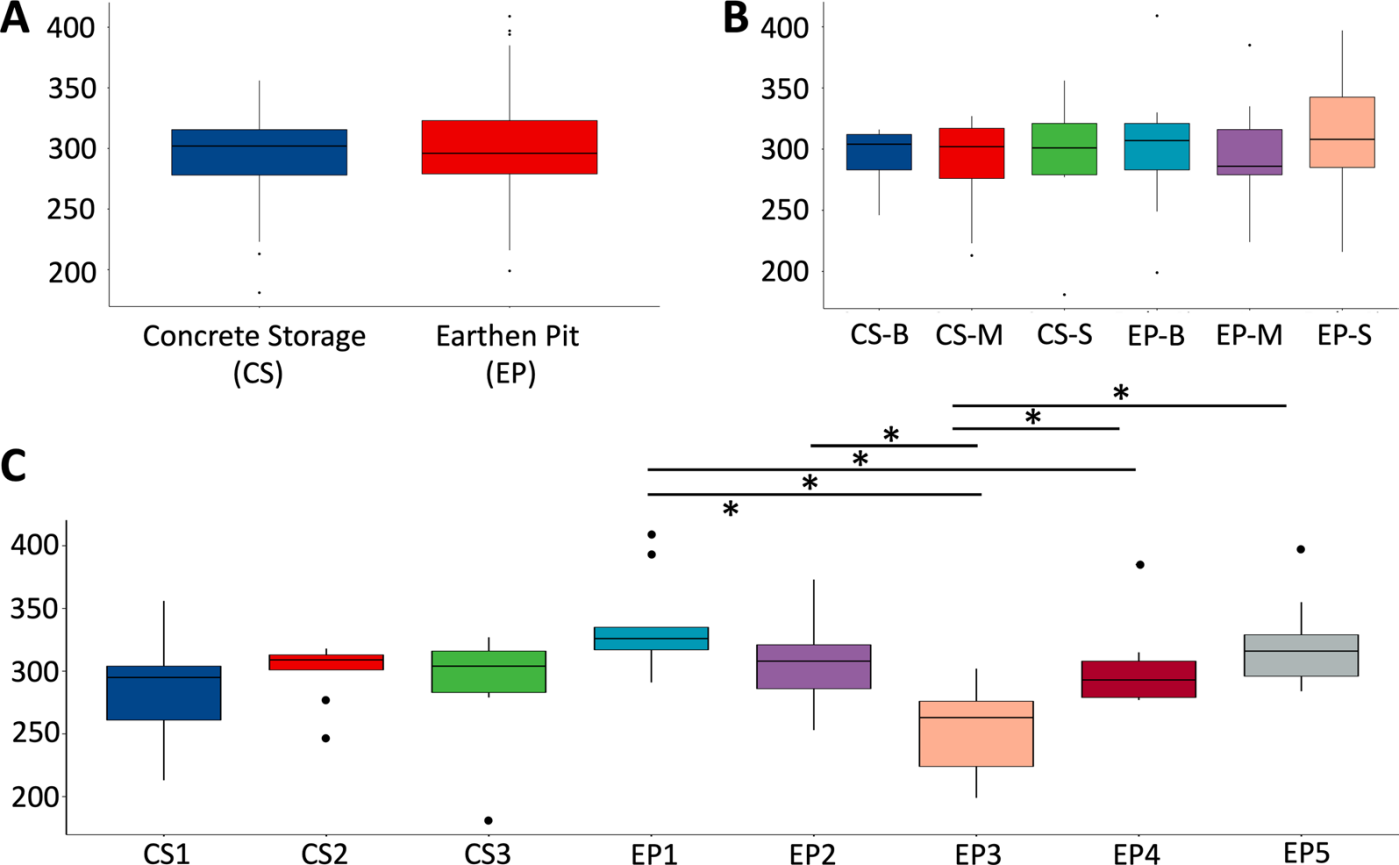
Species richness of the manure microbiome in two storage systems. Overall (A), various depths (B), and various locations (C). Details of the locations and depths are shown in Figs. 1C and F. Significance was calculated with *P*_ANOVA_ < 0.05

Microbial diversities of the microbiomes of the manure stored in EP and CS, as measured in terms of species richness index, were identical (*P*_ANOVA_ > 0.05), although the individual compositions differed (Fig. 3A). Similar results were observed when comparing the microbiomes at various depths in each storage (Fig. 3B). However, this was not the case when comparing microbiomes between the locations within a storage. A significant heterogeneity was observed for microbiome composition between sampling locations in EP (EP1-5, Fig. 3C) (*P*_ANOVA_ < 0.05). Samples collected from EP inlet (EP1) had the most diverse microbial population, followed by those collected from a near outlet location (EP5) (Fig. 3C). The lowest microbiome diversity was observed in the manure samples taken from proximity of the lining (EP3) (Fig. 3C). However, such was not the case with the CS, as the microbiome in this system appeared more uniform over all locations (Fig. 3C).

### Comparing the manure microbiomes of two storage systems

**Fig. 4:**
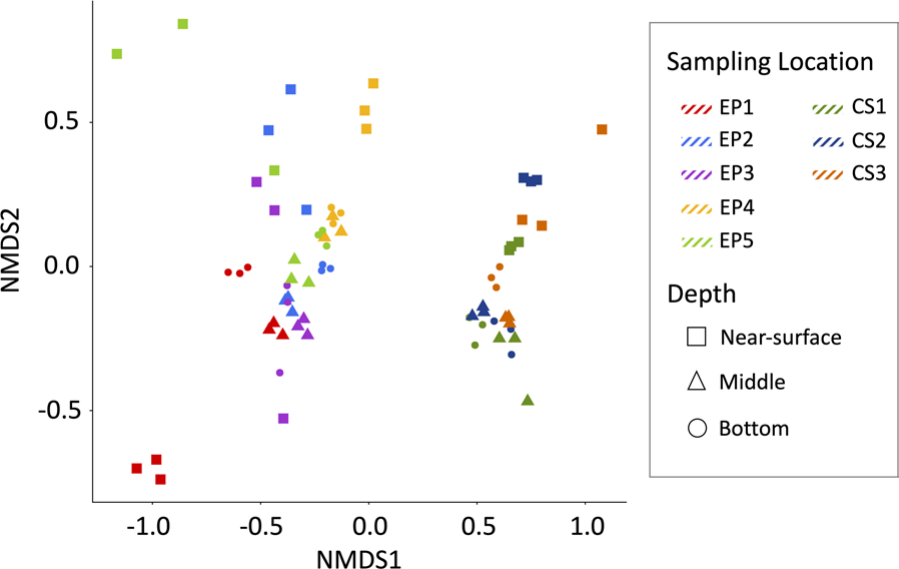
Comparison of the manure microbiome compositions at various locations and depths in two storage systems. The comparison was performed via non-metric multidimensional scaling (nMDS) analysis of the Bray–Curtis distances

In terms of composition, the manure microbiomes of EP and CS displayed a clear separation (Fig. 4). Such separations were also observed between storage depths, with near-surface samples showing the most obvious segregation while the rest were clustered together (Fig. 4). Within the same storage system, EP exhibited higher location-to-location variation in comparison to CS (Fig. 4); the latter showed a tight commonality across all sampling locations. It seems that for EP, the sampling locations near the inlet (EP1) and that with a crust (EP4) were the main drivers of these variations (Fig. 4); as mentioned above, EP1-near-surface and EP4-near-surface samples had substantially higher values for the COD and TS, VS, TKN, ORG-N, TAN and NO_3_^−^-N values than the other sites.

A quantitative assessment of sample parameters that influenced the composition of the stored manure microbiomes was conducted using permutational analysis PERMANOVA and ANOSIM based on Bray Curtis distance matrices with 999 permutation and α-level of 0.05 [36]. The results, presented as the respective P-values in Table 1, revealed that the storage type influenced the microbiome composition in stored dairy manure (*P*_PERMANOVA_ and *P*_ANOSIM_ < 0.05).

**Table 1:** Statistical analysis of the sample parameters

Statistical method	Sample parameter	P-value		R value	
PERMANOVA	Manure storage type, EP and CS	0.001*		0.36	
ANOSIM		0.001*		0.80	
**Statistical method**	**Sample parameter**	**Earthen pit**		**Concrete storage**	
		**P-value**	**R value**	**P-value**	**R value**
PERMANOVA	Sampling location	0.001*	0.37	0.016*	0.15
	Depth	0.003*	0.2	0.001*	0.3
ANOSIM	Sampling location	0.001*	0.37	0.025*	0.11
	Depth	0.001*	0.3	0.001*	0.41

^*^A P-value of < 0.05 in PERMANOVA and ANOSIM analysis identified a sample parameter as a significant factor influencing the microbiome composition (36)

Furthermore, within each storage system, both sampling location and depth contributed to the microbial population structure (Table 1), partially contradicting the results from nMDS analysis which did not identify the sampling location as a driver for sample separation in CS.

**Fig. 5:**
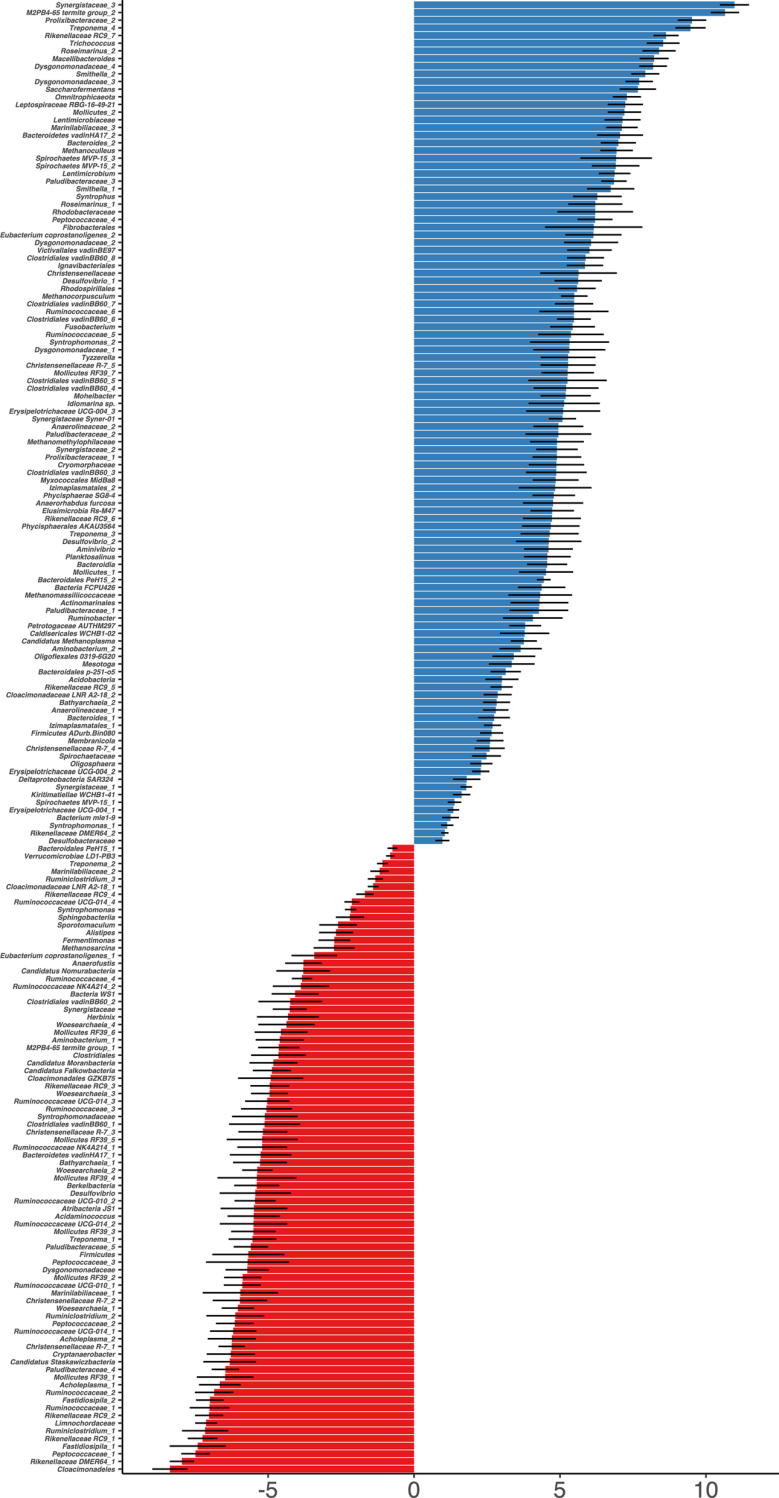
Differential abundance of prokaryotic microorganisms in dairy manure storages. The differentially abundant ASVs were identified using DESeq2 analysis (poscount; significance: *P*_WALD_ < 0.001). Earthen pit (EP), blue; concrete storage (CS), red. X-axis: Average Log2Fold values; Y-axis: assigned lowest taxonomic annotation. Standard error (lfcSE) values are shown as black bars

The ASVs that were more abundant in the EP compared to the CS based on DESeq2 analysis [39], where those having *P*_WALD_ less than 0.001 were classified as enriched. In total, there were 110 enriched ASVs in EP, and 81 in CS (Fig. 5 and Additional file 2: Table S3). Thirteen ASVs representing six Proteobacteria species (*Ruminobacter*, Rhodospirillales, *Rhodobacteraceae*, *Syntrophus*, *Smithella*, and *Desulfovibrio*) were more abundant in EP microbiome, where only one *Desulfovibrio* ASV was enriched in CS (Fig. 5). A similar observation was made for methanogenic members of Euryarchaeota phylum, as 5 ASVs annotated as *Methanophilaceae, Methanomassiliicoccaceae, Methanocorpusculum,* and *Methanoculleus* genera were found in high abundance in EP compared to CS (Fig. 5). A *Methanosarcina* ASV however, was more enriched in CS, followed by other archaeal members from *Nanoarchaeum* (4 ASVs).

While in nMDS clustering the near-surface samples were separated (Fig. 4), the differential analysis using depth as a comparison parameter did not yield a similar observation for this set. In EP, only 2 ASVs representing *Syntrophomonas* and *Ruminococcaceae* were differentially abundant (*P*_WALD_ < 0.001) between the near-surface location and the middle. In contrast, the middle vs bottom comparison identified five differentially abundant ASVs annotated as *Marinilabiliaceae, Hydrogenispora, Herbinix,* and *Cloacimonadales* (Additional file 2: Table S4).

A similar comparison for CS returned 9 and 5 ASVs, respectively (Additional file 2: Table S4). Within these, *Mollicutes* RF39, *Cloacibacillus, Armatimonadetes*, and *Ruminococcaceae* UCG-014 ASVs were found to be more enriched in the middle depth of CS whereas *Ruminofilibacter, Fibrobacter, Treponema, Phycisphaerae* mle1-8, *Ruminiclostridium, Hydrogenospora,* and *Marinilabiliaceae* ASVs were more abundant in the near-surface location. Between the middle and bottom depths of CS, no ASV was found to be significantly abundant, which was concordant with the nMDS analysis results that did not display a sample separation for these sets.

### Microbial community variation by locations in stored dairy manure

**Fig. 6:**
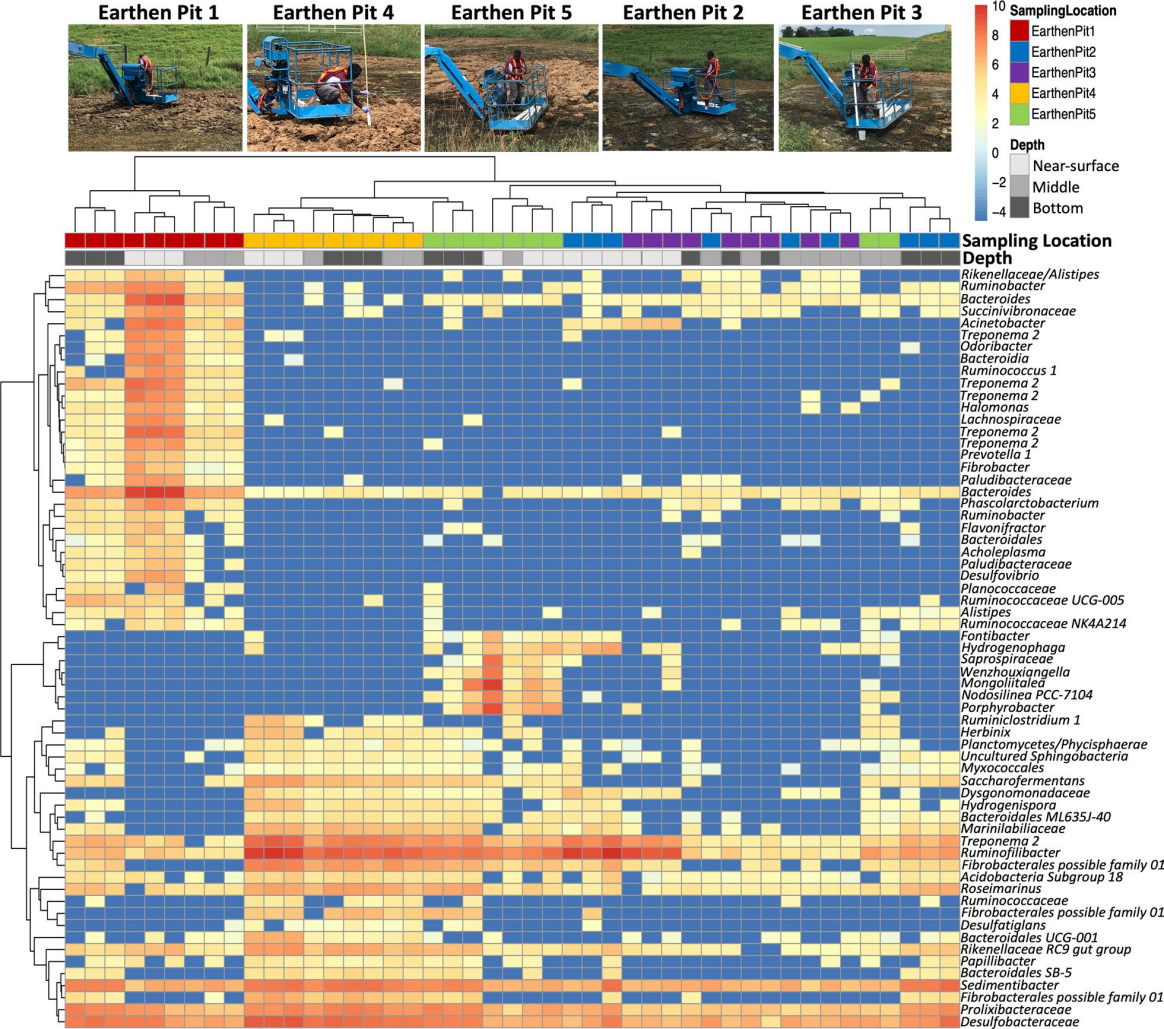
Differentially abundant species of prokaryotic microorganisms at various sites in earthen pit storage. In a DEeq2 analysis, sixty-three ASVs with Wald statistical test value less than 0.001 were defined as significantly differential abundant species. Prior to plotting on a heatmap, the data from these ASVs were normalized using a variance stabilizing transformation algorithm on DESeq2. The lowest taxonomic annotation of the ASVs are shown on the Y-axis

Differential abundance analysis of microbial communities across sampling locations within each storages displayed contrasting results. For example, slight variation was observed over locations in the CS, where heterogeneity was shown only by the enrichment of two ASVs annotated as *Peptococcaceae and Methylophilaceae* in CS1 (center) vs CS3 (inlet); both were more abundant in CS1. In contrast, the EP microbiome displayed more location-to-location variations in composition, as represented by 63 ASVs (Fig. 6 and Additional file 2: Table S5).

The sampling location closest to the inlet (EP1) exhibited the most discrete microbial community (Fig. 6), followed by the EP4 and EP5, and of these only EP4 had a crusted surface. Of the ASVs were detected in the heavily populated EP1 location, 28 had high level similarities to the *Succinivibrionaceae, Acinetobacter, Rikenellaceae, Odoribacter, Halomonas, Paludibacteraceae, Phascolarctobacterium, Flavonibacter, Desulfovibrio,* and *Planococcaceae.* Most of these enriched ASVs were found to be located 0.15 m below the surface (Fig. 6).

In EP, the EP4 and EP5 locations exhibited significantly higher abundance of 26 ASVs (Fig. 6). Some of these ASVs likely represented bacteria from the *Phycisphaerae,* Myxococcales*, Saccharofermentans, Dysgonomonadaceae, Hydrogenispora, Marinilabiliaceae, Roseimanus, Desulfatiglans, Papillibacter, Sedimentibacter, Prolixibacteraceae*, and *Desulfobacteraceae* phyla. In addition, samples originating from the areas near the outlet (EP 5), inlet (EP 2), and storage lining (EP3) shared some commonalities, as shown in the enrichment of 8 ASVs annotated as *Ruminococcaceae* NK4A214*, Fontibacter, Hydrogenophaga, Saprospiraceae, Wenzhouxiangella, Mongoliitalea, Nodosillinea* PCC-7104, and *Porphyrobacter* at these locations (Fig. 6).

### Characterization of nitrogen-transforming microorganisms in manure storage

**Fig. 7:**
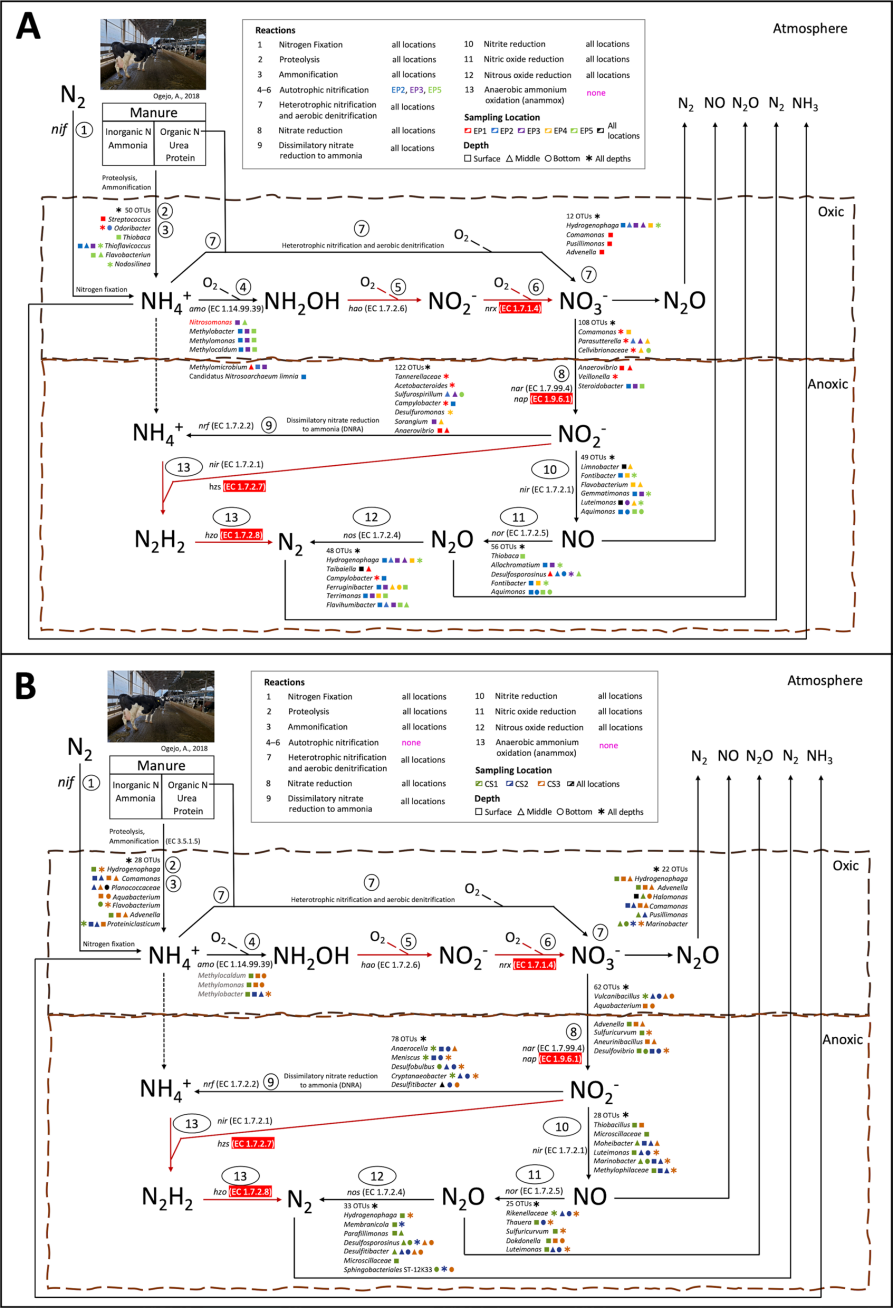
Spatial distribution of nitrogen-transforming and inferred nitrogen transformation processes in two storages. **A** EP, Earthen Pit; **B** CS, Concrete Storage. The relevant transformation processes numbered from 1 to 13 are shown with arrows. Also shown are the lowest taxonomic annotations of the detected ASVs that were assigned to the listed processes. The distribution of the organisms in a storage are displayed with the color-codes for the sampling sites and storage depths. Key genes involved in each process are shown in italics. Genes that were not present in the PICRUSt2 database are highlighted in red

The screening strategy shown in Fig. 2 linked 740 and 430 ASVs (Additional file 2: Tables S6 and S7) to specific nitrogen transformation pathways operating in EP and CS, respectively (Fig. 7). At the next step, we defined their sites of occurrence in the storages and respective relative abundances (Additional file 1: Figs. S2 and S3). With these assignments in hand, the organisms represented by the ASVs with high abundances as well as presence in more than two samples were linked to specific nitrogen transformation processes as shown Figs. 7A and B. Also, the possibility of the occurrences of each nitrogen transformation reaction or pathway at a particular site was also judged based on the respective chemical conditions such as the availability of oxygen that blocks or facilitates certain metabolic processes (Fig. 7).

There were clear possibilities for the microbial production of ammonia in both storages***.*** Many of the organisms represented by the identified ASVs had the enzymatic potentials for degrading protein and nucleic acids, the major nitrogen-containing constituents of cells, and urea, and thereby, producing free ammonia from manure under aerobic and anaerobic conditions; some of these organisms are shown on Reactions 2 and 3 in Fig. 7 and many are listed in Additional file 2: Tables S6 and S7. For example, *Proteiniclasticum*, *Luteimonas*, and *Proteiniphilum* are known to degrade and live on proteins using an inventory of proteases, peptidases and amino acid deaminases [44–46] (Additional file 2: Table S7-8). Similarly, *Pseudomonas*, *Hydrogenophaga, Flavobacterium,* and those from the *Rhodobacteraceae* family could obtain ammonia nitrogen from urea [47–50] (Additional file 2: Tables S6 and S7). As the pH for both storages ranged from 6.92 to 7.85 (Additional file 2: Table S2) and the pKa of ammonia is 9.2, not more than 4% of this compound will occur in the deprotonated or NH_3_ form which could be released to the atmosphere (Additional file 2: Table S2). The ASV data were not analyzed for organisms with nitrogen fixation potentials as manure is rich in fixed nitrogen making nitrogen fixation unlikely to occur in the storages.

We examined the possibilities of microbial conversion of ammonia to non-gaseous and gaseous products. We found that although oxygen could be present at the inlet or in the area immediately underneath the surface, the ASVs detected in both EP and CS did not show a significant representation of the archaea and bacteria that could perform aerobic and autotrophic nitrification. This process occurs either in two steps, nitritation (ammonia nitrite) and nitratation (nitrite nitrate), involving two different organisms, or via a one-step process with one organism that is called comammox (ammonia nitrate) [51–58]. Nitritation is also catalyzed by aerobic ammonia oxidizing archaea and bacteria (AOA and AOB)[51–56, 58]. None of the CS samples carried AOA or AOB ASVs. One of three EP2-near-surface samples harbored an AOA ASV, assigned to Candidatus *Nitrosoarchaeum limnia* (ammonia nitrite) [59] (Fig. 7A), with the relative abundance of 0.03%. For AOB, only one ASV was found in EP. It was annotated as *Nitrosomonas* and associated with two out of 45 samples: one out of three EP3-near-surface samples and one out of three EP5-middle samples with relative abundances of 0.09 and 0.04%, respectively. Consequently, these finding were either artifacts or indicative of an insignificant presence of AOA and AOB in EP. There was no indication of *Nitrospira* species that perform comammox in EP and CS [51, 55–57, 60].

Under limited oxygen concentration, a nitritation function is provided by certain methanotrophs as these bacteria oxidize ammonia to nitrite due to shared structural and functional similarities between ammonia monooxygenase (AMO) and methane monooxygenase (MMO) [61, 62]. Indeed, ASVs representing the methanotrophic species of *Methylocaldum, Methylomonas*, and *Methylobacter* genera [63] were found in both storages. In EP these ASVs were detected exclusively in the near-surface samples at EP2, EP3, and EP5 locations and in CS the respective locations were the near-surface at CS1 and CS3, and the bottom of CS3.

Heterotrophic nitrification (HD) that combines heterotrophic energy production with ammonia oxidation to nitrite and nitrate (ammonia nitrite nitrate) could be coupled to aerobic denitrification (ADN: nitrate nitrite NO N_2_O N_2_) [51–56, 58, 64]. In EP, several ASVs representing the organisms that could catalyze this combined HD-ADN process were found primarily associated with the near-surface samples at multiple locations (shown on reaction 7 in Fig. 7A)[65–68]. In CS, the distribution of such ASVs was mixed with about half being associated with the near-surface locations (reaction 7, Fig. 7B). Thus, in both EP and CS some of the ammonia could be lost, especially from the near-surface locations, through the HD-AND process.

As mentioned above, the input manure for both CS and EP contained nitrate at significant levels (Additional file 2: Table S2). The ASV data presented multiple possibilities for the anaerobic processing of nitrate in the stored manure. Many ASVs (345 in EP and 209 in CS) were linked to organisms that carry nitrate reductase (EC 1.7.99.4). As can be seen in Fig. 7A, each depth (near-surface, middle or bottom) of all locations (EP1-5) of EP likely harbored anaerobic bacteria that together can convert nitrate to N_2_ with intermediary production of NO and N_2_O (Reactions 8 and 10–12, Fig. 7A) [51–56, 58]. A similar situation was observed with CS, except the near-surface regions of most locations (CS1-3). Both EP and CS, exhibited potentials of bacterial dissimilatory reduction of nitrite to ammonia anaerobically (DNRA, Reaction 9, Figs. 7A and B) [51–56, 58] by organisms such as *Campylobacter*, *Geobacter, Meniscus, Opitutaceae,* and *Pelotomaculum* [69–71]. The anammox, an anaerobic denitrification process which couples ammonia oxidation with nitrite reduction producing N_2_, seemed to be absent in the stored manure of EP and CS. This process is catalyzed by *Brocardia*, *Anammoxglobus*, *Scalindua*, *Kuenenia*, and *Jettenia* species which are anaerobic bacteria belonging to the Planctomycetes phylum [72, 73] [74, 75]. The detected ASVs for Planctomycetes did not represent the genera mentioned above but were annotated as species from *Pirellulaceae*, *Phycisphaeraceae*, and *Rubinisphaeraceae* families, none of which are known to perform anammox [72, 76].

### Microbial methane metabolism in stored manure

**Fig. 8:**
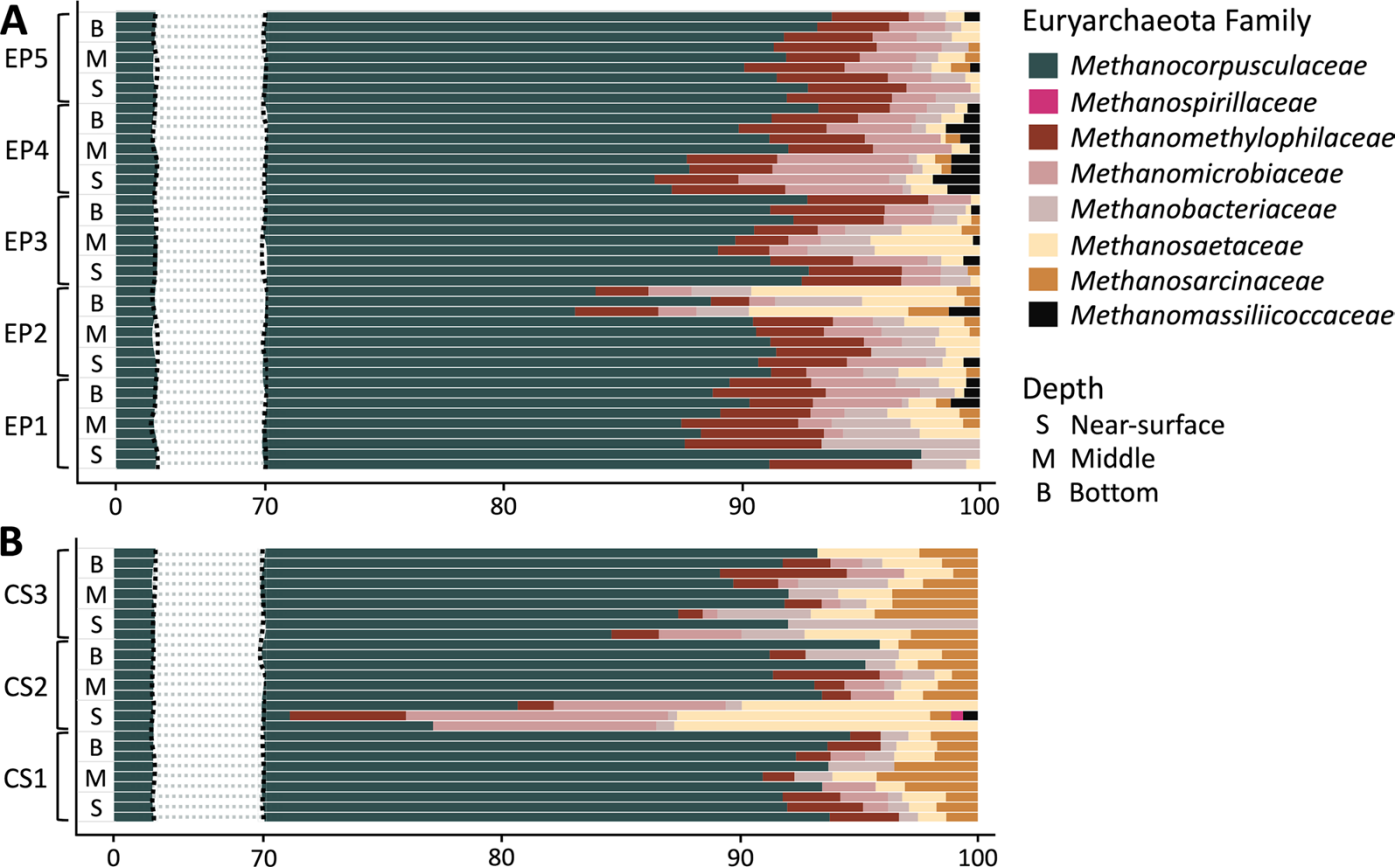
Methanogenic community in stored dairy manure. Average relative abundances of ASVs annotated as Euryarchaeota in earthen pit (EP) and concrete storage (CS) at various sampling sites and depths are shown. The locations of EP1-5 and CS1-3 are shown in Fig. 1

The ASVs representing methanogens were detected in both storages at average relative abundances of 7.73% and 5.95% for EP and CS, respectively. *Methanocorpusculaceae,* a hydrogenotrophic methanogen family, comprised up to 95% of the Euryarchaeota sequences for both storage systems (Fig. 8 and Additional file 2: Table S8). Other observed families were *Methanosaetaceae, Methanosarcinaceae, Methanomethylophilaceae*, and *Methanomicrobiaceae* (Fig. 8). For the low-abundance families, EP and CS differed substantially, as detected counts of the members of *Methanomethylophilaceae* and *Methanomassiliicoccaceae* were higher in EP and that of *Methanosarcinaceae* were higher in CS. The anaerobic methane oxidizing archaea, which are close relatives of methanogens [77, 78], were not found in the samples analyzed.

**Fig. 9:**
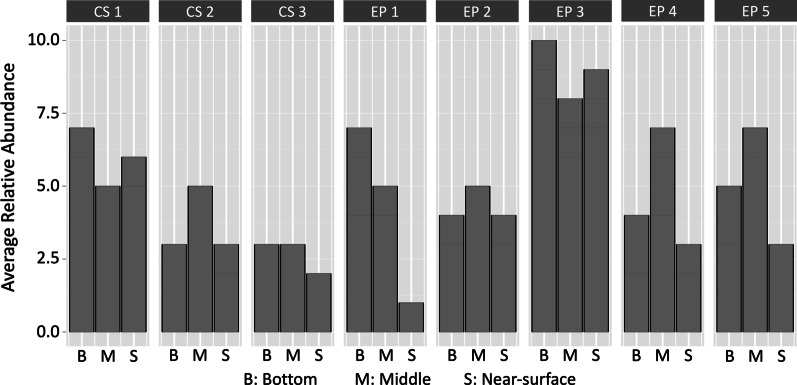
Abundance of *Methanocorpusculaceae* in stored dairy manure. Average relative abundance of 3 ASVs annotated as *Methanocorpusculaceae* in earthen pit (EP) and concrete storage (CS) at various sampling sites and depths are shown. The locations of EP1-5 and CS1-3 are shown in Fig. 1

The results of Kruskal–Wallis and Wilcoxon rank test revealed that the difference between the relative abundances of Euryarchaeota in the two storages was significant (Additional file 2: Table S9). However, this was not the case when the comparison was between the sampling sites and depths within the same storage (Additional file 2: Table S9). In EP, the location close to the lining of the storage (EP3) was found with the highest methanogen relative abundance. In contrast, in CS it was the center of the storage (CS1) that had this characteristic (Fig. 9). In a comparison across storage depths in EP, the inlet location (EP1) exhibited maximum variations. For this location, the highest methanogen prevalence was found at the bottom, and from there, it was progressively lower towards the middle and near-surface locations (Fig. 9). For other locations in EP and CS, little variation in methanogen prevalence was observed among the depths.

## Discussion

We have characterized the microbiomes of dairy manure stored in an EP and a CS in two commercial dairy farms for their potential to transform nitrogen into soluble and volatile inorganic species. First, we identified the archaeal and bacterial ASVs that occurred in the stored manure by analyzing the determined sequences of the V4 regions of the 16S rRNA genes. Then we assigned the potentials for catalyzing various nitrogen transformation reactions to these prokaryotes. With that information, we developed models for the pathways that allow the stabilization and loss of nitrogen in EP and CS. We have also determined the diversity of the detected methane-forming archaea or methanogens and developed concepts for their relative impacts on methane production in these two manure storage systems. We elaborate below on these findings and their importance in analyzing the performances of manure storage systems in small commercial dairy farms.

The nature of microbial nitrogen metabolism in the two storage types investigated appeared to be determined by the complex and anaerobic nature of the manure. The presence of organic nitrogen helped enrich an abundance of prokaryotic organisms with the capability of generating ammonia from the complex nitrogenous compounds and urea. Oxygen can penetrate maximum up to a depth of 7–10 cm beneath the surface of stored manure [79], creating a strictly anoxic environment in most areas of this system. Consequently, the autotrophic nitrifiers that require oxygen were almost absent in the stored manure, whereas anaerobic heterotrophic nitrifiers were present abundantly (Fig. 7). Such selections are also known to be favored by a high C/N ratio present in manure [80]. While autotrophic nitrifiers are extremely sensitive towards acidic pH [24, 81], this factor was not responsible for their absence as the pH of manure in both storages was in the neutral to slightly alkaline range (6.92 – 7.85) (Additional file 2: Table S2). For the above-mentioned environmental status, it was also unlikely that ammonia was lost from the stored manure via a combined action of autotrophic or heterotrophic nitrifiers and aerobic denitrifiers (Reactions 4–7, Fig. 7).

In contrast with the situation described above, anaerobic respiration driven denitrification (Reactions 8 and 10–12, Fig. 7) provided a route through which the microbiomes of both EP and CS could have emitted NO, N_2_O and N_2_ via the transformation of nitrate. The input manure contained nitrate at appreciable concentrations, up to 6–12 times that of the stored manure (Unpublished data, Jactone Arogo Ogejo, 2022). This was likely a product of aerobic microbial processes, such as Reaction 7 of Fig. 7A, that occurred on the cattle barn floor before manure was scrapped off to the storage. This high concentration is the likely reason for the observed high diversity and abundance of nitrate reducers at the inlet area of both storages (EP1 and CS3, Fig. 7). A diversion from the denitrification process catalyzed by the bacteria that perform dissimilatory nitrite reduction to ammonium (DNRA) (Reaction 9, Fig. 7), presented a possible way of retaining some of the NO_3_^−^-N in the stored manure. The ASV data yielded a curious observation, an apparent absence of the anammox process in both systems. As nitrite is the limiting substrate for this reaction (82–84), the possible reasons to the absence of anammox are high flux operations of the respiratory denitrification (Reactions 10–12, Fig. 7) and DNRA (Reaction 9, Fig. 7) or the absence of or poorly functioning anaerobic nitrate reduction process (Reaction 8, Fig. 7). It is also possible that chosen 16S rRNA primer set was not able to capture the anammox community [85, 86]. In future studies, this problem could be mitigated by use of the *hydrazine oxidoreductase* gene (*hzo*) as additional marker which has been proven to be effective in capturing the presence and diversity of anammox bacteria better [83].

In the context of above-mentioned general possibilities, EP carried more nitrogen transformation associated ASVs (Additional file 2: Table S6). It also exhibited substantial site to site variations, which was limited in CS. At a given sampling location of either EP or CS, the composition of the manure microbiome at 0.15 m below the surface was distinct from those in the middle and bottom, and the latter two were similar (Figs. 4 and 7). This separation was likely due to oxygen exposure to the near-surface location and uniform anaerobic conditions further down. Except for this variation, the CS established a nearly common microbiome composition at all locations, whereas EP offered variations by location. We hypothesize that this distinction arose from the differences in the design that led to distinct chemical and structural characteristics of the storages, such as the solid and N-content, and surface crusting. The CS was made up of cylindrical concrete tank with concrete floor, with no contact with adjoining environment except that the top was open to the air. In contrast, the EP was oval shaped with a clay lining, which could allow permeation of aqueous solutions with soluble organic and inorganic components from the adjoining soil into the storage. This storage could also receive soil parts including respective microbes. In CS, manure was added to surface at a peripheral location (CS3, Fig. 1F), and in EP, the addition occurred at the bottom of a similar location (EP1, Fig. 1C). Thus, it is also possible that manure moved from the point of entry to the exit area via two distinct flow paths in these two storages. It was likely uniform in all directions in CS whereas in EP there was an indication of an ununiform movement of manure or locally distinct microbiome activities. In fact, this case was exemplified in the microbiome composition and chemical conditions at the EP4 location as discussed below.

In EP, the site near the inlet (EP1) provided the highest species richness (Fig. 3) and distinct microbiome comprised of 563 ASVs. This status was likely due to the freshest input material that floated to the surface, which provided the EP-near-surface location with the highest levels of TS, VS, TKN, ORG-N, NO_3_^−^-N and TAN compared to other locations, and perhaps a minor amount of oxygen that was introduce to this site by the addition system. While these observations with EP1 were reasonable, the situation with the EP4, which was located midway between the inlet (EP1) and the outlet (EP5), showcased an unusual nature of EP. Compared to EP1, the EP4 location harbored microbiomes of distinct compositions (Fig. 6). These unusual characteristics were consistent with the prevailing physiochemical conditions at the location. The TS, VS, TKN, ORG-N, and TAN levels at EP4 were higher than those at EP2, EP3 and EP5 and similar to the values seen at EP1 (Additional file 2: Table S2). The surface of EP4 also carried a crust which likely developed from the drying of the foam generated by gas bubbles arising from the bottom and carrying undigested plant fibers of manure. This incidence was indicative of a more active gas producing anaerobic degradation activity at this site. Some of the microbiome characteristics of EP4 were seen at EP5 (Fig. 6).

Since the manure storages have been reported to have potential to lose up to 30% of the total nitrogen [10] and the microbial metabolism did not seem to be a major driver for such a major loss, we hypothesize that it is a combination of physiochemical processes that accounts for a majority of the loss. As mentioned about, at the prevailing pH of 6.92 to 7.85 of the manure, EP and CS will maintain less than 4% of the ammonia in the volatile NH_3_. However, as the vapor is blown away by wind, the system would generate more NH_3_ to maintain the equilibrium and causing substantial loss of ammonia from the storage. This hypothesis is consistent with the observations that the loss of total nitrogen from EP could rise fourfold if the wind speed increases from 0 to 5 m per hour and the presence of up to 30 cm crust reduces ammonia emission or nitrogen loss by twofold [87].

All of the Euryarchaeota ASVs detected in EP and CS corresponded to the methanogens (Fig. 8). These communities were dominated by the *Methanocorpusculum* species (Fig. 8–9), an observation that has been previously reported for manure storages [4, 5, 20]. Since these methanogens are hydrogenotrophs [88], the methane emission from a manure storage would be tightly linked to the hydrogen production by fermentative bacteria [89]. Significant abundance of *Methanomethylophilaceae* and *Methanomassiliicoccaceae* ASVs were observed in EP while CS had more prevalence of *Methanosarcina* (Fig. 8). This is a major contrast in terms of the methanogenesis from methyl group containing substrates, as *Methanomethylophilaceae* and *Methanomassiliicoccaceae* are obligately dependent on hydrogen for the reduction of methyl groups to methane and do not use other methanogenic substrates, whereas *Methanosarcinaceae* make methane from methylotrophic substrates with and without hydrogen and can use several other substrates for methane production [90, 91]. It is possible that with higher abundance and diversity of methanogen (Fig. 9 and Additional file 2: Table S7), EP was a higher methane emitter than CS. However, it should be noted that the 16S rRNA copy numbers and the abundance of a particular type of methanogens are not always true indicators of a higher methane production activity of a methanogenic system [19].

## Conclusions

The study assessed the composition of nitrogen transforming and methanogenic prokaryotic communities in two types of dairy manure storages (EP and CS), and in the process it tested the hypothesis that this feature is influenced by the storage type. It was found that in general, EP and CS provided similar metabolic outcomes and EP was distinguished for its site-to-site variations. In both cases, while the microbes detected therein will generate ammonia from proteins, nucleic acids and other complex organic compounds and urea, they will not oxidize this product to soluble or gaseous nitrogenous compounds. There was a possibility that the nitrate generated through chemical or microbial oxidation occurring in the manure on the barn floor would be converted to NO, N_2_O and N_2_, which are gases, through a denitrification process. A likely route for converting nitrate and preserving it as ammonia was also detected. Thus, the microbial processes were not the likely drivers for the reported loss of nitrogen from the storages and a shift in the equilibrium towards the volatilization of ammonia due to removal of this compound by wind was the likely cause. The crust that forms on manure could counter this effect.

The earthen pit storage (EP) established a more complex ecosystem with greater location to location compositional heterogeneity than CS, and this distinction was likely due to an ununiform movement of manure and interactions with the adjoining soil areas in the EP, which CS did not offer. The production of methane in both storages was likely driven primarily by the species that could utilize the hydrogen generated from the fermentative degradation of the complex carbon compounds of manure. In EP, even the methane production from methyl group containing compounds was performed by methanogens that are dependent on hydrogen.

The microbiomes of both storages had the potential of generating greenhouse gases such as methane, NO, and N_2_O. With a higher abundance of methanogens, EP could be a higher producer of methane and here a location near the lining had a more potential for this activity. A rapid removal of manure from the barn floor, and thereby, lowering the production of nitrate, could reduce NO and N_2_O emission from these storages and methane production could be reduced with a better isolation of the earthen pit storage from the adjoining soil.

Our results clearly revealed a complex nature of commercial manure storage systems in terms of their microbiomes. As mentioned in the introduction, there is a lack of detailed studies on the relationships between the microbiome metabolism and retention of nitrogen fertilizer and greenhouse gas emission in manure storage systems of small dairy farms. This is a serious concern as the designs of such storages are not fully similar to those studied in research laboratories, and therefore, the results from the latter may not be able to predict the outcomes for the former well. A need for a better understanding of the nitrogen transformation processes occurring in the manure on the barn floor was also identified. These gaps prevent the development of meaningful whole farm nutrient accounting models. Thus, the current study, which relies on 16S rRNA amplicons, provides motivation for more detailed investigations with more incisive approaches such metagenomics including the generation of metagenome-assembled genomes (MAGs), metatranscriptomics, metaproteomics, metabolomics, and metabolic modeling, leading to predictive models for the storage outcomes and better designs for the manure storages.

## Supplementary information

**Additional file 1.** Custom built manure sample collection system, site-specific ASV heatmaps, and supplementary DNA extraction method.

**Additional file 2.** Supplementary Tables.

## Data Availability

The raw sequences of the 16S rRNA-V4 amplicons have been deposited in NCBI SRA under the BioProject ID PRJNA842411.
