# Supplementary material for: Nitrogen transformation processes catalyzed by manure microbiomes in earthen pit and concrete storages on commercial dairy farms
Source: Environ Microbiome. 2023 Apr 11;18:32. doi: 10.1186/s40793-023-00483-z (PMC10091836; doi:10.1186/s40793-023-00483-z)
Supplement: Supplementary file 1 — Additional file 1. Custom built manure sample collection system, site-specific ASV heatmaps, and supplementary DNA extraction method. [file 40793_2023_483_MOESM1_ESM.docx]

**Nitrogen Transformation Processes Catalyzed by Manure Microbiomes in Earthen Pit and Concrete Storages on Commercial Dairy Farms**

**Supplementary File 1**

Bela Haifa Khairunisa^1^, Usha Loganathan^2^, Jactone A. Ogejo^3#^, Biswarup Mukhopadhyay^1,2#*^

^1^Genetics, Bioinformatics, and Computational Biology Ph.D. Program, Virginia Tech, Blacksburg, VA 24061, USA

^2^Department of Biochemistry, Virginia Tech, Blacksburg, VA 24061, USA

^3^Department of Biological System Engineering, VA 24061, USA

#Correspondence should be addressed to Jactone A. Ogejo (arogo@vt.edu) and Biswarup Mukhopadhyay (biswarup@vt.edu).

*Review contact corresponding author: Biswarup Mukhopadhyay; [biswarup@vt.edu](mailto:biswarup@vt.edu)

**Figure S1.** **Custom built manure sample collection system.** PVC and CPVC pipes (Charlotte Pipe & Foundry Company, NC)**_._ PVC-1**, 1-½-in diameter SCH40, 5-ft length (Cat. no: PVC 07112 1000); **PVC-2**, ¾-in diameter SCH40, 10-ft length (Cat. no: PVC 04007 0600); **CPVC**, ½-in diameter and 5-ft length (Cat. no: CTS 12005 0500); **PCC**: Sharkbite ½ in push-to-connect coupling (Model no. U008LFZ, Sharkbite, GA); **SCV (valve)**, S-613 Sump check valve with stainless steel bands, 1-½-in diameter and 25 Psi pressure rating (Cat. no: 203-227, Legend Valve, MI).


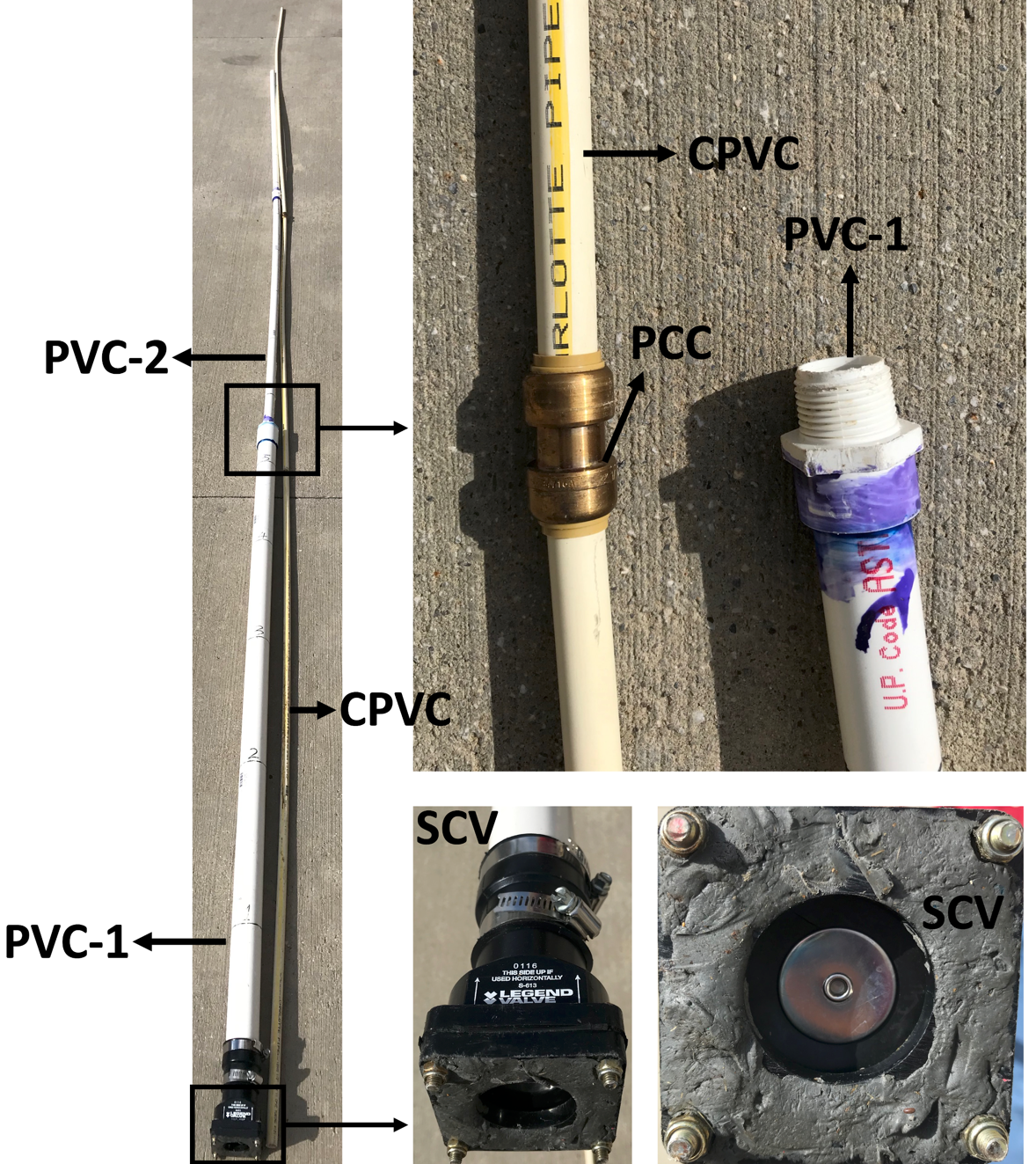


The sampler was operated as follows. The CPVC pipe was inserted into the PVC-1-PVC-2 assembly through the open top end of PVC-2 and pushed onto the SCV (*valve*) to keep it closed. Then the entire assembly was lowered into the stored manure, maintaining the pressure of the CPVC on the valve to keep the latter shut. Once the lower or the valve end of the assembly reached the desired depth, CPVC was lifted a bit to open the valve, letting the sample to enter into PVC-1. After allowing a bit of time for the sample to enter, the CPVC was pushed down to close the valve. With the valve closed the assembly was pulled out from the manure, and the sample was allowed to flow out into a beaker by opening the valve.

**Figure S2. Site-specific enrichment of ASVs assigned to nitrogen transformation process in the earthen pit storage (EP).** VST-transformed values of 740 ASVs that are potentially involved in nitrogen transformation during manure storage are presented as heat map, using the scale as shown on the right of the figure. Sampling location and depths, as defined in Fig. 1, are shown on the X-axis and Y-axis presents the ASV ids.


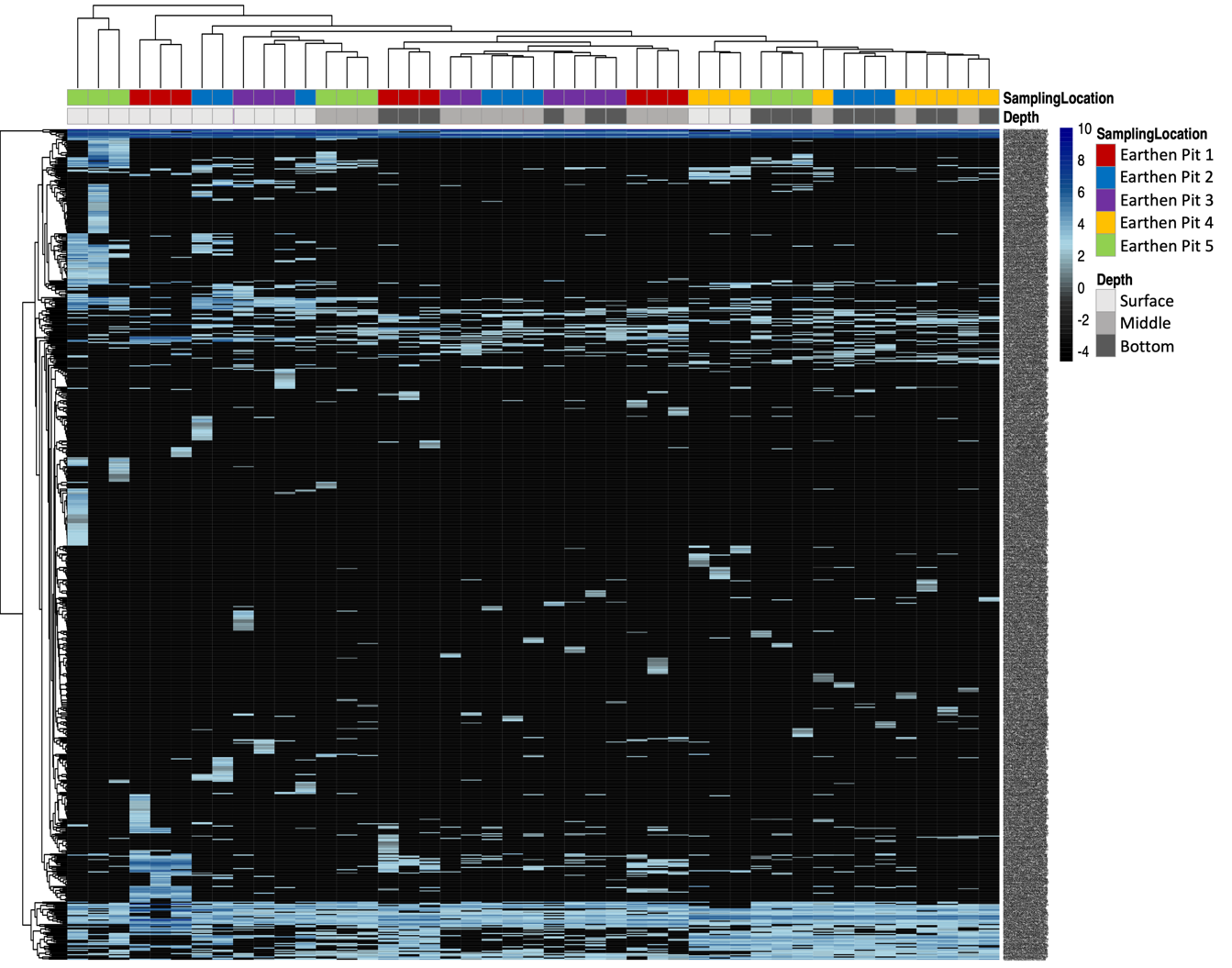

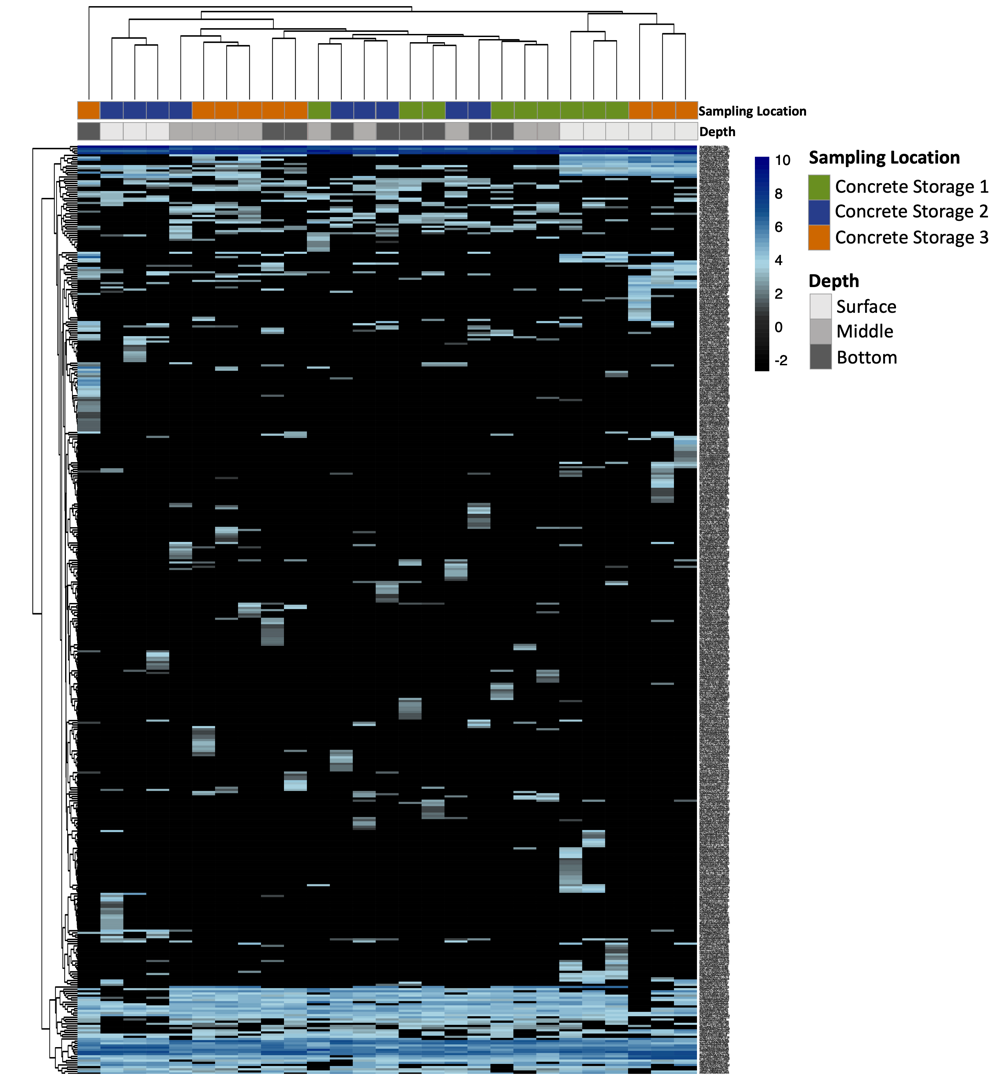


**Figure S3. Site-specific enrichment of ASVs assigned to nitrogen transformation process in the concrete storage (CS).** VST-transformed values of 270 ASVs that are potentially involved in nitrogen transformation during manure storage are presented as heat map, using the scale as shown on the right of the figure. Sampling location and depths, as defined in Fig. 1, are shown on the X-axis and Y-axis presents the ASV ids.

**Supplementary Method**

Method S1. DNA extraction

In brief, 200 mg of a manure sample was mixed with 1 ml of inhibitEX buffer (cat. no. 19593, Qiagen, Germantown, MD) in a 1.5 ml polypropylene microcentrifuge tube and the mixture was vortexed vigorously for one minute using a standard heavy-duty vortex mixer (VWR International, LCC, Radnor, PA) operating at 2,500 rpm. The homogenized sample was incubated at 90°C for 5 minutes, vortexed again for 15 seconds at 2,500 rpm, and centrifuged at 16,000 x g for one minute. The resultant supernatant was transferred into a new microcentrifuge tube and the centrifugation step was repeated twice to minimize solid material carry over. To the clarified supernatant, RNase A (Sigma-Aldrich; cat. no. 9001-99-4) was added to a final concentration of 0.5 mg/ml. The mixture was incubated at 37°C for 3 minutes and then centrifuged for 3 minutes at 16,000 x g. Resulted aqueous phase was transferred to a fresh microcentrifuge tube and mixed with Qiagen Proteinase K to a final concentration of 0.83 mg/ml. Then, an equal volume of Buffer AL (Qiagen) was added to the mixture. The rest of the steps followed the manufacturer’s instruction (Qiagen). Quantification and quality assessment of DNA samples were performed via both agarose gel electrophoresis and Nanodrop measurements (NanoDrop Lite UV-Vis Spectrophotometer; cat. no. ND-LITE; ThermoFisher Scientific, Waltham, MA). In order to determine whether a DNA preparation contained inhibitors that could affect downstream processes, full length 16S rRNA genes were PCR amplified using universal primers 27F and 1525R (1). Samples yielding a full-length product were considered suitable for sequencing.

**Reference**

1. Lane DJ. 1991. 16S/23S rRNA Sequencing, p 115-175. *In* Stackebrandt E, Goodfellow M (ed), Nucleic Acid Techniques in Bacterial Systematic. John Wiley and Sons, New York.
